# Linking Plasma Amyloid Beta and Neurofilament Light Chain to Intracortical Myelin Content in Cognitively Normal Older Adults

**DOI:** 10.3389/fnagi.2022.896848

**Published:** 2022-06-17

**Authors:** Marina Fernandez-Alvarez, Mercedes Atienza, Fatima Zallo, Carlos Matute, Estibaliz Capetillo-Zarate, Jose L. Cantero

**Affiliations:** ^1^Laboratory of Functional Neuroscience, Pablo de Olavide University, Seville, Spain; ^2^Network Center for Biomedical Research in Neurodegenerative Diseases (CIBERNED), Madrid, Spain; ^3^Departamento de Neurociencias, Achucarro Basque Center for Neuroscience, Universidad del País Vasco, Leioa, Spain; ^4^Ikerbasque, Basque Foundation for Science, Bilbao, Spain

**Keywords:** aging, Alzheimer’s disease, intracortical myelin, functional connectivity, blood biomarkers, amyloid-beta, neurofilament light

## Abstract

Evidence suggests that lightly myelinated cortical regions are vulnerable to aging and Alzheimer’s disease (AD). However, it remains unknown whether plasma markers of amyloid and neurodegeneration are related to deficits in intracortical myelin content, and whether this relationship, in turn, is associated with altered patterns of resting-state functional connectivity (rs-FC). To shed light into these questions, plasma levels of amyloid-β fragment 1–42 (Aβ_1–42_) and neurofilament light chain (NfL) were measured using ultra-sensitive single-molecule array (Simoa) assays, and the intracortical myelin content was estimated with the ratio T1-weigthed/T2-weighted (T1w/T2w) in 133 cognitively normal older adults. We assessed: (i) whether plasma Aβ_1–42_ and/or NfL levels were associated with intracortical myelin content at different cortical depths and (ii) whether cortical regions showing myelin reductions also exhibited altered rs-FC patterns. Surface-based multiple regression analyses revealed that lower plasma Aβ_1–42_ and higher plasma NfL were associated with lower myelin content in temporo-parietal-occipital regions and the insular cortex, respectively. Whereas the association with Aβ_1–42_ decreased with depth, the NfL-myelin relationship was most evident in the innermost layer. Older individuals with higher plasma NfL levels also exhibited altered rs-FC between the insula and medial orbitofrontal cortex. Together, these findings establish a link between plasma markers of amyloid/neurodegeneration and intracortical myelin content in cognitively normal older adults, and support the role of plasma NfL in boosting aberrant FC patterns of the insular cortex, a central brain hub highly vulnerable to aging and neurodegeneration.

## Introduction

Aging is the major risk factor for Alzheimer’s disease (AD), but the reasons why aging increases susceptibility to AD are poorly understood. One contributing factor may be the perturbation of myelin-related genes (Mathys et al., 2019) that eventually leads to widespread degeneration of myelin sheaths. This phenomenon results in slowing of conduction velocity along nerve fibers, modifying the timing of network oscillations, and ultimately affecting functional connections within neural circuits (Peters, 2002). The efficiency of remyelination also declines with age, likely due to the limited regenerative capacity of oligodendrocyte progenitor cells (Irvine and Blakemore, 2006). Consequently, lightly myelinated axons become more vulnerable to irreversible degeneration during aging, favoring cognitive decline (Wang et al., 2020) and the spread of AD pathology before the onset of symptoms (Mitew et al., 2010; Braak and Del Tredici, 2015; Couttas et al., 2016; Dean et al., 2017; Luo et al., 2019).

New ultrasensitive quantitative technologies allow the identification of proteins in blood at subfemtomolar concentrations (Rissin et al., 2010; Yang et al., 2017), opening new avenues for the development of blood biomarkers capable of detecting individuals at risk for cognitive decline and AD. Accumulated evidence suggests that lower levels of plasma amyloid-β fragment 1–42 (Aβ_1–42_) are associated with accelerated aging and AD. Accordingly, low levels of plasma Aβ_1–42_ at baseline have shown to increase the risk of cognitive decline (Seppälä et al., 2010), mild cognitive impairment (MCI) (Rembach et al., 2014) and AD (Rembach et al., 2014; Chouraki et al., 2015; Hilal et al., 2018; de Wolf et al., 2020). Moreover, higher plasma concentrations of neurofilament light chain (NfL), the main cytoskeletal structure of myelinated axons, have been associated with increased brain Aβ load in cognitively unimpaired older adults (Chatterjee et al., 2018; Benedet et al., 2020), and have shown to predict cortical thinning and subsequent cognitive impairment in the preclinical stage of both familial and sporadic AD (Preische et al., 2019; Lee et al., 2022). However, it remains largely unknown whether plasma markers of amyloid and neurodegeneration bear any relation to brain myelin content in cognitively normal older individuals, which may be relevant to establish surrogate markers of vulnerability to AD.

Although myelination is a prominent feature of the subcortical white matter (WM), the gray matter (GM) of the cerebral cortex also contains a substantial amount of myelinated axons unevenly distributed in layers (Nieuwenhuys, 2013). Thus, myelin density is higher in deeper than in superficial cortical layers and in sensorimotor than in association regions (Nave and Werner, 2014). This particular distribution of cortical myelin may partially account for the functional organization of the neocortex (Beul et al., 2017; Huntenburg et al., 2017). Moreover, evidence has shown that subtle changes in myelin have meaningful effects on neuronal network function (Waxman, 1980; Felts et al., 1997; Pajevic et al., 2014). Therefore, aging-related patterns of rs-FC may be driven by cortical regions showing myelin deficits, which, in turn, may be conditioned by plasma markers of amyloid and neurodegeneration.

Here, we specifically addressed these questions by investigating whether the T1w/T2w ratio, considered as a proxy of myelin content in the cortical GM (Glasser and Van Essen, 2011), is associated with plasma levels of Aβ_1–42_ and NfL in cognitively normal older adults, and whether this relationship varies with cortical depth. Next, we assessed whether those cortical areas showing plasma measurements-related myelin deficits also exhibited alterations in their pattern of rs-FC, which in turn may account for the variability in cognitive functioning. Therefore, the present study sought to test three inter-related hypotheses. As amyloid pathology has been previously associated, either direct or indirectly, with myelin damage (Desai et al., 2010; Mitew et al., 2010; Schmued et al., 2013; Dean et al., 2017; Luo et al., 2019; Chen et al., 2021), we first hypothesized that lower levels of plasma Aβ_1–42_ will be associated with lower intracortical myelin content in cognitively normal older adults. The relationship between aging-related axonal damage and myelin breakdown is likely strengthened by dysfunctional oligodendrocytes, which supply energy for axonal metabolism (Mot et al., 2018). Accordingly, our second prediction was that higher levels of plasma NfL will be accompanied by lower myelin content, and that this relationship will become more evident in inner cortical layers, where oligodendrogenesis is most impaired (Orthmann-Murphy et al., 2020; Xu et al., 2021). Finally, and given the critical role of myelin in neuronal communication and fine-tuning of neuronal circuits (Fields, 2015; Monje, 2018), we postulated that cortical regions showing lower myelin content related to plasma levels of Aβ_1–42_ and/or NfL will also show altered patterns of rs-FC.

## Materials and Methods

### Participants

One hundred thirty-three cognitively normal older adults participated in the study (65 ± 5.9 years; range: 54–76 years; 81 females). They were recruited from senior citizen’s associations, health-screening programs, and hospital outpatient services. All of them underwent neurological and neuropsychological assessment to discard both the presence of dementia and objective cognitive impairment. Individuals with medical conditions that affect brain structure or function (e.g., cerebrovascular disease, epilepsy, head trauma, history of neurodevelopmental disease, alcohol abuse, hydrocephalus, and/or intracranial mass) were not included in the study. Participants met the following criteria: (i) normal global cognitive status in the Mini-Mental State Examination (scores ≥ 26); (ii) normal cognitive performance in the neuropsychological tests relative to appropriate reference values for age and education level; (iii) global score of 0 (no dementia) in the Clinical Dementia Rating; (iv) functional independence as assessed by the Spanish version of the Interview for Deterioration in Daily Living Activities (Böhm et al., 1998); (v) scores ≤5 (no depression) in the short form of the Geriatric Depression Scale (Sheikh and Yesavage, 1986); and (vi) not be taking medications that affected cognition, sleep, renal or hepatic function. All participants gave informed consent to the experimental protocol approved by the Ethical Committee for Clinical Research of the Junta de Andalucía according to the principles outlined in the Declaration of Helsinki. Table 1 contains sample characteristics.

**TABLE 1:** Demographics, cognitive and biochemical variables.

Age	65 ± 5.9
Sex (F/M)	81/52
Education years	11.7 ± 4.5
ApoE4 (yes/no)	31/102
MMSE	28.6 ± 1.3
Memory Binding Test	
Total free recall	16.2 ± 4.6
Pairs in free recall	5.3 ± 2.6
Total paired recall	27.1 ± 4.9
Paired recall pairs	9.8 ± 4.1
Total delayed free recall	15.1 ± 5.0
Pairs in delayed free recall	5.8 ± 3.9
Total delayed paired recall	28.9 ± 5.4
Boston Naming Test	12.1 ± 2.1
Phonological fluency	15.7 ± 4.4
Semantic fluency	22.0 ± 17.0
Trail Making Test-A	47.0 ± 21.5
Trail Making Test-B	119.5 ± 67.7
Tower of London	319.3 ± 113.2
Plasma Aβ_1–42_ (pg/ml)	12.1 ± 5.1 (2.4 – 24)
Plasma NfL (pg/ml)	12.9 ± 7.2 (2.3 – 39.6)

*Results are expressed as mean ± SD, unless otherwise stated. F/M, females/males; MMSE, Mini Mental State Examination; NfL: neurofilament light.*

### Neuropsychological Assessment

All participants received neuropsychological assessment. They were administered with the following tests: the Spanish version of the Memory Binding Test (MBT) (Gramunt et al., 2016); the short form of the Boston Naming Test (BNT); the semantic and phonological fluency tests based on the “Animal” and letter “P” naming tasks; the two forms of the Trail Making Test (TMT-A and TMT-B); and the Tower of London (TL). All neuropsychological scores were z transformed. In the case of TMT-A and TMT-B, we used the inverse z-values, while in the case of the MBT and TL we first computed a composite measure by summing the z-scores of the different scores. We then applied principal component analysis to obtain the Spearman’s “*g*” factor as an index of global cognitive function. This analysis was done with R Statistical Software v3.0.1 (R Foundation for Statistical Computing, Vienna, Austria) using the *prcomp* function. We only retained the first component (eigenvalue 3.4), which explained 53.7% of variance in the data, due to the contribution of MBT (24.2), BNT (24.2), phonological fluency (24.2), semantic fluency (3.2), TMT-A (8.6), TMT-B (9.5), and TL (6.0). The standardized residuals were used to obtain the latent variable *g* (factor loading MBT: 0.92; BNT: 0.92; phonological fluency: 0.92; semantic fluency: 0.33; TMT-A: 0.55; TMT-B: 0.57; TL: 0.46).

### Measurements of Plasma Aβ_1–42_ and NfL

Fasting blood samples were taken at 9:00-10:00 AM in all participants to control for potential circadian effects. Briefly, venous blood samples were collected in 10 ml dipotassium ethylene diamine tetraacetic acid (EDTA) coated tubes (BD Diagnostics), and immediately centrifuged (1,989 *g*) at 4°C for 5 min. Supernatant plasma was aliquoted into polypropylene tubes containing 300 μl of plasma mixed with 10 μl of a protease inhibitor cocktail (cOmplete Ultra Tablets mini, Roche), and stored at –80°C until analysis. Plasma samples used in the present study were not previously thawed.

Plasma Aβ_1–42_ and NfL levels were measured on the ultra-sensitive single-molecule array (Simoa) HD-1 analyzer platform (Quanterix, MA, United States) following the manufacturer’s instructions. The Aβ_1–42_ Simoa 2.0 assay (Cat. No. 101664) and the NF-light Simoa assay advantage kits (Cat. No. 103186) were purchased from Quanterix. These assays measure Aβ_1–42_ and NfL levels in human plasma with a detection limit of 0.044 and 0.038 pg/ml, respectively. Two quality control samples were run on each plate for each analyte. Plasma Aβ_1–42_ and NfL determinations were run in duplicates, and the average of the two measurements (pg/ml) was used for statistical analysis. Samples with coefficients of variation higher than 20% were excluded.

### Magnetic Resonance Imaging Acquisition

Structural and functional magnetic resonance imaging (MRI) scans were performed on a 3T Philips Ingenia MRI scanner, equipped with a 32-channel radio-frequency (RF) receive head coil and body RF transmit coil (Philips, Best, Netherlands). The following MRI sequences were acquired in the same session: (i) 3D T1-weighted (T1w) magnetization prepared rapid gradient echo (MPRAGE) in the sagittal plane: repetition time (TR)/echo time (TE) = 2,600 ms/4.7 ms, flip angle (FA) = 9°, acquisition matrix = 384 × 384, voxel resolution = 0.65 mm^3^ isotropic, resulting in 282 slices without gap between adjacent slices; (ii) 3D T2w scan in the sagittal plane: TR/TE: 2,500 ms/251 ms, FA = 90°, acquisition matrix = 384 mm × 384 mm, voxel resolution = 0.65 mm^3^ isotropic, resulting in 282 slices without gap between adjacent slices; and (iii) T2w Fast Field Echo images using a blood-oxygen-level-dependent (BOLD) sensitive single-shot echo-planar imaging (EPI) sequence in the axial plane: TR/TE: 2,000 ms/30 ms, FA = 80°, acquisition matrix = 80 mm × 80 mm, voxel resolution = 3 mm^3^ isotropic, resulting in 35 slices with 1 mm of gap between adjacent slices. To allow for optimal B1 shimming, a B1 calibration scan was applied before starting the EPI sequence. We acquired 250 EPI scans preceded by four dummy volumes to allow time for equilibrium in the spin excitation. Before starting the acquisition of the EPI sequence, participants were asked to remain still and keep their eyes closed without falling sleep. Pulse and respiratory rates were recorded using the scanner’s built-in pulse oximeter placed on the left-hand index finger and a pneumatic respiratory belt strapped around the upper abdomen, respectively. Brain images were visually examined after each MRI sequence; they were repeated if artifacts were clearly identified. All participants underwent the same MRI protocol at the MRI facility of Pablo de Olavide University.

### Structural MRI Preprocessing and Generation of Cortical Myelin Maps

T1w scans were preprocessed using Freesurfer v6.0.^1^ The Freesurfer’s pipeline included brain extraction, intensity normalization, automated tissue segmentation, generation of white matter (WM) and pial surfaces, correction of surface topology and inflation, co-registration, and projection of cortical surfaces to a sphere for establishing a surface-based coordinate system (Fischl and Dale, 2000). Pial surface misplacements and erroneous WM segmentation were manually corrected on a slice-by-slice basis by one experienced technician. The T2w image was registered to the T1w image with *bbregister* employing a trilinear interpolation method. *bbregister* is a within-subject, cross-modal registration program that uses a boundary-based cost function constrained to 6 degrees of freedom (Greve and Fischl, 2009).

We have used the T1w/T2w ratio image to indirectly estimate the relative intracortical myelin content in individual cortical surfaces. Previous studies have shown that regional variations in the T1w/T2w ratio match the myelin content derived from histologically-defined cortical areas (Glasser and Van Essen, 2011) and they further correlate with cortical myelination patterns across the lifespan (Grydeland et al., 2019). This is partially due because myelin alters the signal intensity of T1w and T2w images in opposite directions and, consequently, the T1w/T2w ratio provides both enhanced tissue contrast and sensitivity to brain myelin content (Uddin et al., 2019). Furthermore, the T1w/T2w ratio cancels radiofrequency (RF) receive field (B1−) artifacts in the absence of head motion, and corrects reasonably well for RF transmit field (B1+) effects when the participants’ head is precisely located at the isocenter of the magnetic field (Glasser et al., in press). In the present study, the latter requirements were strictly met to reduce transmit field biases and spurious results in cortical myelin maps.

**FIGURE 1:**
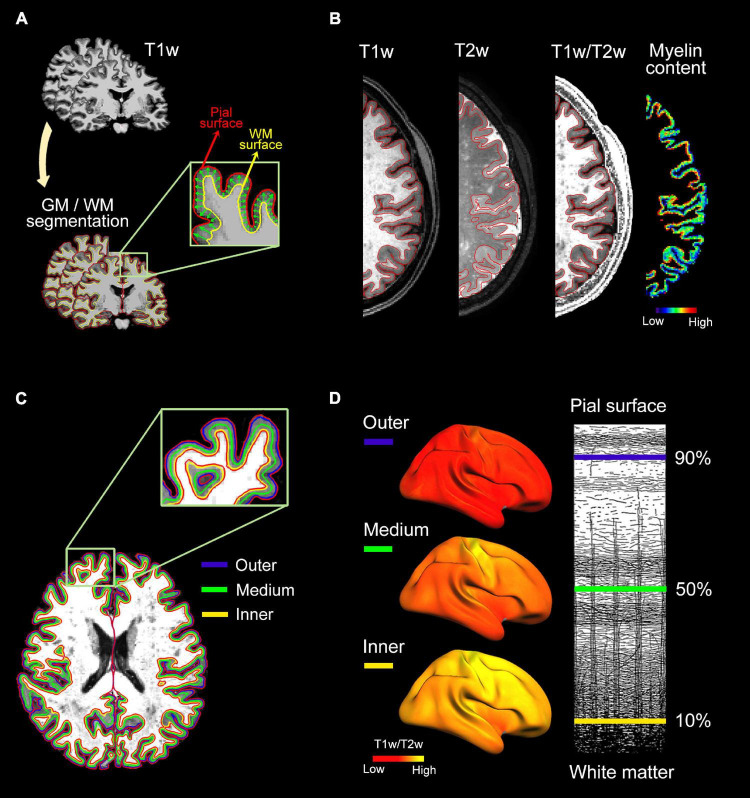
Analysis pipeline for intracortical myelin mapping. (A) Individual T1w scans were skull-stripped. Next, pial and WM boundaries were established using semi-automatic segmentation procedures implemented in Freesurfer. (B) Intracortical myelin content was obtained by computing the voxel-wise ratio of T1w to T2w. Warm colors represent regions of high intracortical myelin while cold colors represent regions of low myelin content, as revealed by T1w/T2w ratio values. (C) Three uniformly spaced layers were delineated within the cortical ribbon of the T1w/T2w ratio image: 90% (outer layer), 50% (medium layer) and 10% (inner layer) of the cortical thickness away from the WM surface. (D, Left panel) Individual myelin maps obtained at different cortical depths were projected onto the average cortical surface. (Right panel) Illustrative representations of the three cortical depths superimposed on myeloarchitectonic organization of the human neocortex (Figure adapted from Vogt, 1903).

The tissue fraction effect was corrected in individual T1w/T2w ratio images using PETsurfer (Greve et al., 2016), setting the point spread function estimate to zero. Next, three intermediate surfaces were generated within the cortical GM at fixed relative distances between the WM and pial surfaces (Polimeni et al., 2010). These surfaces were located at 90% (outer layer), 50% (medium layer) and 10% (inner layer) of the cortical thickness away from the WM surface. Finally, individual myelin maps obtained at different cortical depths were projected onto the average cortical surface, transformed to z-scores, and smoothed using non-linear spherical wavelet-based de-noising schemes (Bernal-Rusiel et al., 2008). All processing steps were visually checked for quality assurance. Figure 1 illustrates the analysis pipeline for intracortical myelin mapping.

### Functional MRI Preprocessing and Resting State-Functional Connectivity Analysis

Resting-state functional magnetic resonance imaging (rs-fMRI) data were preprocessed using AFNI functions,^2^ version AFNI_20.3.01. For each participant, high-frequency spikes were eliminated (*3dDespike*), time-locked cardiac and respiratory motion artifacts on cerebral BOLD signals were minimized using RETROICOR (Glover et al., 2000), time differences in slice-acquisition were corrected (*3dTshift*), EPI scans were aligned using rigid body motion correction and selecting the first volume as reference (*3dVolreg*), and aligned EPI scans were co-registered to their corresponding T1w volumes (*3dAllineate;* cost function: lpc + ZZ).

Dynamics were removed provided that more than 5% of voxels exhibited signal intensities that deviated from the median absolute deviation of time series (*3dToutcount*); and/or when the Euclidean norm (*enorm*) threshold exceeded 0.3 mm in head motion. None of the participants showed more than 20% of artifactual dynamics after applying censoring. Simultaneous regression was further applied to minimize the impact of non-neuronal fluctuations on the rs-fMRI signal (*3dTproject*). Nuisance regressors included: (i) six head motion parameters (3 translational and 3 rotational) derived from the EPI scan alignment along with their first-order derivatives, (ii) time series of mean total WM/CSF signal intensity, and (iii) cardiac (measured by pulse oximeter) and respiratory fluctuations plus their derivatives to mitigate effects of extracerebral physiological artifacts on cerebral BOLD signals. No temporal band-pass filtering was applied.

Preprocessed rs-fMRI scans were projected onto the *fsaverage5* cortical surface space. Seeds for FC analyses were derived from cortical regions showing significant associations between plasma measurements (i.e., Aβ_1–42_ and NfL levels) and intracortical myelin content. Surface-based rs-FC seed to whole cortex maps were obtained using the Fisher’s z-transform of the corresponding Pearson’s correlation coefficients.

### Sample Size Estimation

To estimate the sample size, we performed power analysis with the G*Power software (v3.1.9.6).^3^ Only two studies have previously assessed the relationship between Aβ_1–42_ and intracortical myelin content, one in the AD continuum using both CSF Aβ_1–42_ and amyloid PET (Luo et al., 2019) and other in cognitively normal older adults using amyloid PET (Yasuno et al., 2017). To our knowledge, research investigating associations between NfL levels and intracortical myelin content or addressing the potential moderating effect of plasma measurements (i.e., Aβ_1–42_ or NfL) on the relationship between intracortical myelin content and cortical rs-FC patterns is lacking. Therefore, we computed an *a priori* (prospective) power analysis (fixed model, R^2^ deviation from zero) to achieve statistical power of 80% given a 0.025 two-sided significance level and an overall Cohen’s effect size (f^2^) ranging from 0.05 to 0.2. Supplementary Figure 1 shows the results of this analysis for additive models evaluating the relationship between one of the plasma measurements (either Aβ_1–42_ or NfL) and intracortical myelin content (3 predictors), and for interactive models assessing the moderating effect of one of the aforementioned plasma measurements on the relationship between intracortical myelin and rs-FC (5 predictors). This analysis revealed that 263 and 308 participants are required to detect an overall effect size of 0.05 for additive and interactive models, respectively. As the study sample consisted of 133 participants, interpretation of significances is conditional on the existence of overall effect sizes equal to or greater than 0.10 and 0.12 for main and interaction effects, respectively.

### Statistical Analysis

We first applied the frequentist approach to determine whether age was related to plasma measurements and intracortical myelin after adjusting for the effects of sex. Next, by applying the same approach, we performed vertex-wise multiple linear regression analyses to evaluate the relationship of plasma measurements (either Aβ_1–42_ or NfL) with intracortical myelin content at different cortical depths (90, 50, and 10% away from the WM surface), including age and sex as covariates of no interest. To assess whether associations between plasma measurements and intracortical myelin content differed with cortical depth, we first computed the mean of the sum of all significant regions at the three cortical depths to extract the fitted values. These fitted values were entered into a mixed model with subjects as random effect to determine whether the strength of these associations varied significantly as a function of cortical depth. The effect of the moderator factor (i.e., cortical depth) was evaluated through an ANOVA that compared the null model only including the intercept with the experimental model including cortical depth as a fixed effect. If differences reached significance, *post hoc* comparisons were performed with the R package emmeans and multiple comparisons were adjusted using the Bonferroni correction.

Using the frequentist approach, we further evaluated whether cortical regions showing significant associations with plasma measurements also exhibited altered patterns of rs-FC. For this, we applied a vertex-wise multiple regression model that included as regressor of interest the interaction between plasma measurements (either Aβ_1–42_ or NfL) and the mean intracortical myelin of the region used as seed in the rs-FC analysis. These models were adjusted by age and sex.

Vertex-wise regression analyses for intracortical myelin and rs-FC were performed using the SurfStat package.^4^ Results were corrected for multiple comparisons using a hierarchical statistical model that first controls the family-wise error rate at the level of clusters by applying random field theory over smoothed statistical maps (*p*_vertex_ < 0.001, *p*_cluster_ < 0.05), and next controls the false discovery rate at the level of vertex within each cluster (*p* < 0.05) over unsmoothed statistical maps (Bernal-Rusiel et al., 2010). The anatomical location of significant results was based on vertices showing the maximum statistic within each significant cluster (Desikan et al., 2006).

After inferential evidence of a main or an interaction effect, we computed the standardized measure of effect size (i.e., Cohen’s f^2^) to evaluate local effect size within the context of a multivariate regression model (Cohen, 1988). To establish the precision of standardized effect sizes, we computed 95% confidence intervals (CI_95%_) using the normal approximated interval with bootstrapped bias and standard error (*N* = 10,000 bootstrap samples) with the Matlab’s *bootci* function.

Next, for each cortical vertex showing the maximum statistic within each significant cluster, we applied Bayesian linear regression analyses using JASP, version 0.12.2.^5^ The Bayesian approach allowed us to quantify the evidence for the alternative hypothesis and to overcome the problem of multiple comparisons resulting from performing different models for two hemispheres and three cortical depths. Bayesian linear regression analyses were based on non-informative priors such as the Jeffreys-Zellner-Siow prior with an r scale of 0.354 (Liang et al. (2008). We compared the strength of the Bayes factor for the model including all covariates of no interest (null model) with the model including the predictor of interest (experimental model) (BF_10_). The classification scheme proposed by Lee and Wagenmakers (2013) was employed to interpret the BF_10_. We only reported those results that reached significance according to the frequentist approach as long as the evidence in favor of the alternative hypothesis was at least moderate (BF_10_ ≥ 3).

Finally, we evaluated whether associations of intracortical myelin content with either plasma Aβ_1–42_/NfL levels or rs-FC accounted for the variability in global cognitive function. For this purpose, we first applied the Yeo-Johnson transformation to the cognitive index (i.e., the latent variable *g*) to reduce the detrimental effects of skewedness and heteroscedasticity in the different models (Yeo and Johnson, 2000). Next, we built four models to assess main and interaction effects. The first model only included the covariates of no interest; the second model the main effects; the third model the two-way interactions between the different predictors of interest; and the fourth model the three-way interaction. We first calculated F-tests between the different sequential models, and then compared the relative strength of the Bayes factor between the models.

## Results

### Associations of Age With Intracortical Myelin and Plasma Levels of Aβ_1–42_ and NfL

Age was positively related to plasma Aβ_1–42_ (F_1_,_130_ = 9.8, *p* = 0.002) and NfL levels (F_1_,_130_ = 28.8, *p* < 10^–6^). On the contrary, age was negatively associated with intracortical myelin content in postcentral regions of the left hemisphere (F_1_,_130_ = 21.2, *p* < 10^–5^) and superior parietal lobe of the right hemisphere (F_1_,_130_ = 21.3, *p* < 10^–5^). These results are illustrated in Supplementary Figure 2.

### Relationship Between Intracortical Myelin and Plasma Levels of Aβ_1–42_ and NfL

**FIGURE 2:**
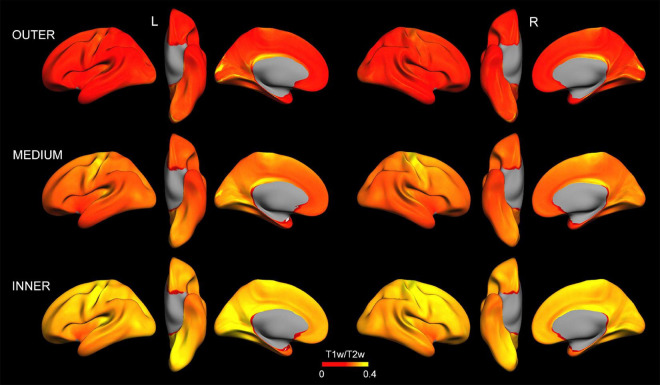
Intracortical myelin maps projected onto the average cortical surface obtained at different depths (outer, medium, and inner layer) before z-transformation. Note that, in general, T1w/T2w ratio values increase with cortical depth. Left (L) and right (R).

**FIGURE 3:**
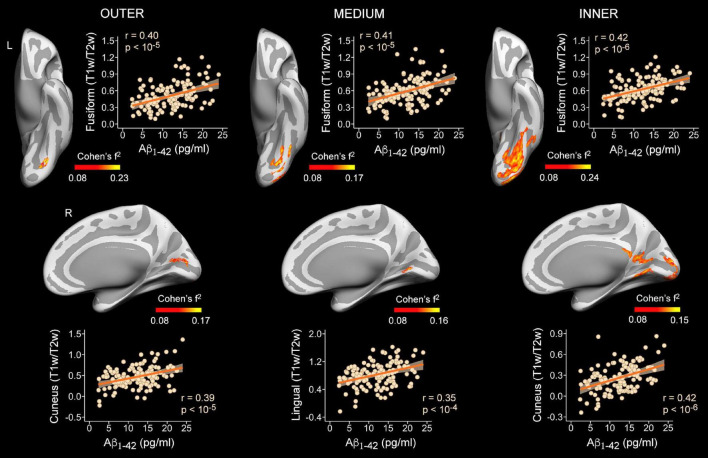
Associations between plasma Aβ_1–42_ levels and intracortical myelin content (T1w/T2w ratio values) on cortical surfaces obtained at 90% (outer layer), 50% (medium layer) and 10% (inner layer) of the cortical thickness away from the WM surface. Projected size effects on inflated cortical surfaces indicate positive associations of plasma Aβ_1–42_ levels with intracortical myelin content, after adjusting for age and sex. The color bar indicates the range of overall size effects (Cohen’s f^2^). Left (L) and right (R). Scatter plots show partial correlations between plasma Aβ_1–42_ levels and the mean myelin content of the most significant cluster for each cortical depth and hemisphere, adjusted by age and sex.

Figure 2 displays intracortical myelin maps projected onto the average cortical surface obtained at different depths (i.e., outer, medium and inner layers) before z-transformation. As expected, T1w/T2w ratio values increased with cortical depth (Nieuwenhuys, 2013; Nave and Werner, 2014). Our T1w/T2w ratio maps differ from those reported in Glasser and Van Essen (2011), especially in some regions of the occipital cortex, whose intracortical myelin content was associated with plasma levels of Aβ_1–42_ (see Figure 3). These differences may be due to the distinct approaches used to obtain the intracortical myelin maps in the two studies. Thus, we used absolute values comprising the entire range of T1w/T2w ratio values instead of a percentile scaling between 3 and 96%, as in Glasser and Van Essen (2011). Neither we adjusted the color palette to identify the transitions between adjacent areas, since we were not interested in delineating spatial gradients or myeloarchitectural features of cortical regions (Glasser and Van Essen, 2011).

Plasma levels of Aβ_1–42_ were positively associated with T1w/T2w ratio values in temporo-parietal-occipital regions of both hemispheres at the three cortical depths, as illustrated in Figure 3 and reported in Table 2. The evidence in favor of the alternative hypothesis was extreme (BF_10_ > 100) for myelin content of inner and outer layers, and moderate in the medium layer (BF_10_ was reported in Table 2).

**TABLE 2:** Significant associations of plasma Ab_1–42_ and NfL with T1w/T2w ratio intensity values.

Vertex location with maximum statistic	MNI	R^2^	F_5_,_127_	$f_{\text{local}}^{2}$	CI_95%_	BF_10_
***Positive associations with A*β*_1–42_ (inner layer)***						
L fusiform (*p*_cluster_ = 10^–6^)	–40 –73 –14	0.18	27.3	0.50	0.47–0.54	220^E^
L lateral occipital (*p*_cluster_ = 10^–5^)	–30 –90 –15	0.19	34.7	0.53	0.50–0.56	1234^E^
L inferior temporal (*p*_cluster_ = 0.02)	–42 –51 –11	0.12	16.4	0.28	0.25–0.32	87^VS^
R lateral occipital (*p*_cluster_ = 10^–6^)	19 –85 –8	0.11	18.3	0.35	0.32–0.38	2436^E^
R precuneus (*p*_cluster_ = 0.0004)	13 –54 16	0.13	19.0	0.38	0.35–0.42	6145^E^
R lingual (*p*_cluster_ = 0.003)	7 –60 2	0.13	19.4	0.35	0.32–0.38	2047^E^
***Positive associations with A*β*_1–42_ (medium layer)***						
L fusiform (*p*_cluster_ = 0.0003)	–30 –72 –16	0.14	13.2	0.31	0.28–0.34	3.9^M^
R lingual (*p*_cluster_ = 10^–5^)	4 –62 7	0.14	16.7	0.36	0.32–0.39	4.2^M^
***Positive associations with A*β*_1–42_ (outer layer)***						
L fusiform (*p*_cluster_ = 10^–5^)	–40 –71 –18	0.19	13.2	0.34	0.31–0.37	1817^E^
L superior parietal (*p*_cluster_ = 0.0002)	–23 –84 24	0.14	18.8	0.37	0.34–0.40	2643^E^
R pericalcarine (*p*_cluster_ = 10^–5^)	5 –78 12	0.12	5.5	0.34	0.31–0.37	7472^E^
R lateral occipital (*p*_cluster_ = 10^–5^)	20 –89 –8	0.15	21.3	0.36	0.33–0.39	3547^E^
***Negative associations with NfL (inner layer)***						
R insula (*p*_cluster_ = 0.04)	41 0 4	0.16	25.4	0.46	0.42–0.49	3.4^M^

*MNI coordinates are in MNI152 space. f^2^: measure of local effect size. CI_95%_: 95% confidence interval. BF_10_: Bayes factor yielded by the Bayesian linear regression analysis. The superscript of the BF_10_ indicates the qualitative interpretation of the evidence for the alternative hypothesis: ^M^ moderate; ^VS^ very strong; ^E^ extreme. L: left; R: right.*

**FIGURE 4:**
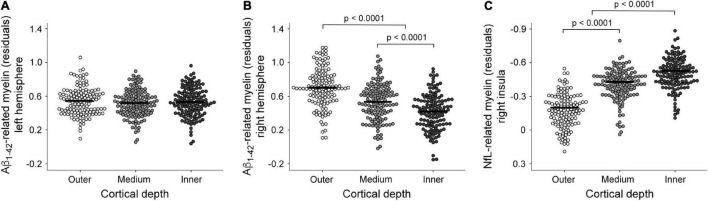
Boxplots showing individual fitted mean intracortical myelin values for regions showing a significant association with plasma measurements at different cortical depths. Effect of cortical depth for the Aβ_1–42_ associations with intracortical myelin in the left **(A)** and right hemisphere **(B)**. Note that positive associations between plasma Aβ_1–42_ levels and intracortical myelin content (T1w/T2w ratio values) increased from inner to outer cortical layers only in the right hemisphere. **(C)** Effect of cortical depth for the NfL associations with intracortical myelin of the right insula. Note that the association between plasma NfL levels and intracortical myelin content (T1w/T2w ratio values) was more negative in the inner than in the medium and outer layers.

Regarding the association between Aβ_1–42_ and intracortical myelin, mixed model ANOVAs revealed a significant main effect of cortical depth for the right hemisphere (χ^2^ = 346.6, *p* < 10^–15^) that was not extended to the left hemisphere (χ^2^ = 5.7, *p* = 0.06). *Post hoc* analyses showed that the strength of positive associations in the right hemisphere decreased with cortical depth, being stronger for the outer than for the other two layers (*p* < 0.0001), and stronger for the medium than for the innermost layer (*p* < 0.0001). Figures 4A,B illustrate these results.

**FIGURE 5:**
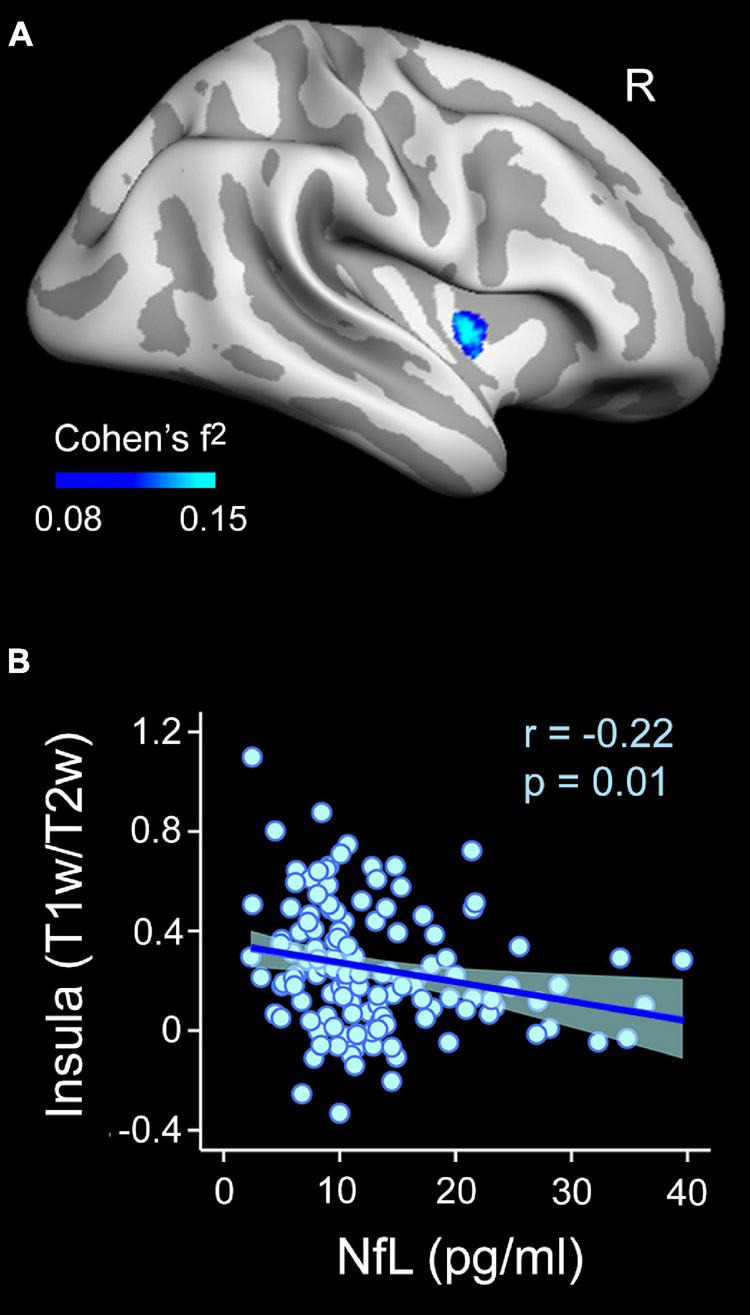
Associations between plasma NfL levels and intracortical myelin content (T1w/T2w ratio values) on the cortical surface obtained at 10% of the cortical thickness away from the WM surface (inner layer). **(A)** Projected size effects on the inflated cortical surface indicate negative associations of plasma NfL levels with intracortical myelin content, adjusted by age and sex. The color bar indicates the range of overall size effects (Cohen’s f^2^). Right (R). **(B)** The scatter plot shows the partial correlation between plasma NfL levels and the mean myelin content of the significant cluster for the inner cortical layer located in the right insula, adjusted by age and sex.

The regression analysis also revealed a negative association between plasma NfL levels and T1w/T2w ratio values of the right insular cortex (Figure 5 and Table 2). This result was restricted to the inner layer, where the Bayesian linear regression analysis showed a BF_10_ of 3.4, indicating that the alternative hypothesis is about three times more likely than the null, which is classified as moderate. The mixed model ANOVA addressing the effect of cortical depth on the strength of this association revealed a significant main effect in the right insular cortex (χ^2^ = 354.9, *p* < 10^–15^). The association of NfL with intracortical myelin was more negative for the innermost layer than for the other two layers (*p* < 0.0001). Figure 4C illustrates these results.

### Effect of the Relationship Between Plasma Measurements and Myelin Content on Rs-FC

**FIGURE 6:**
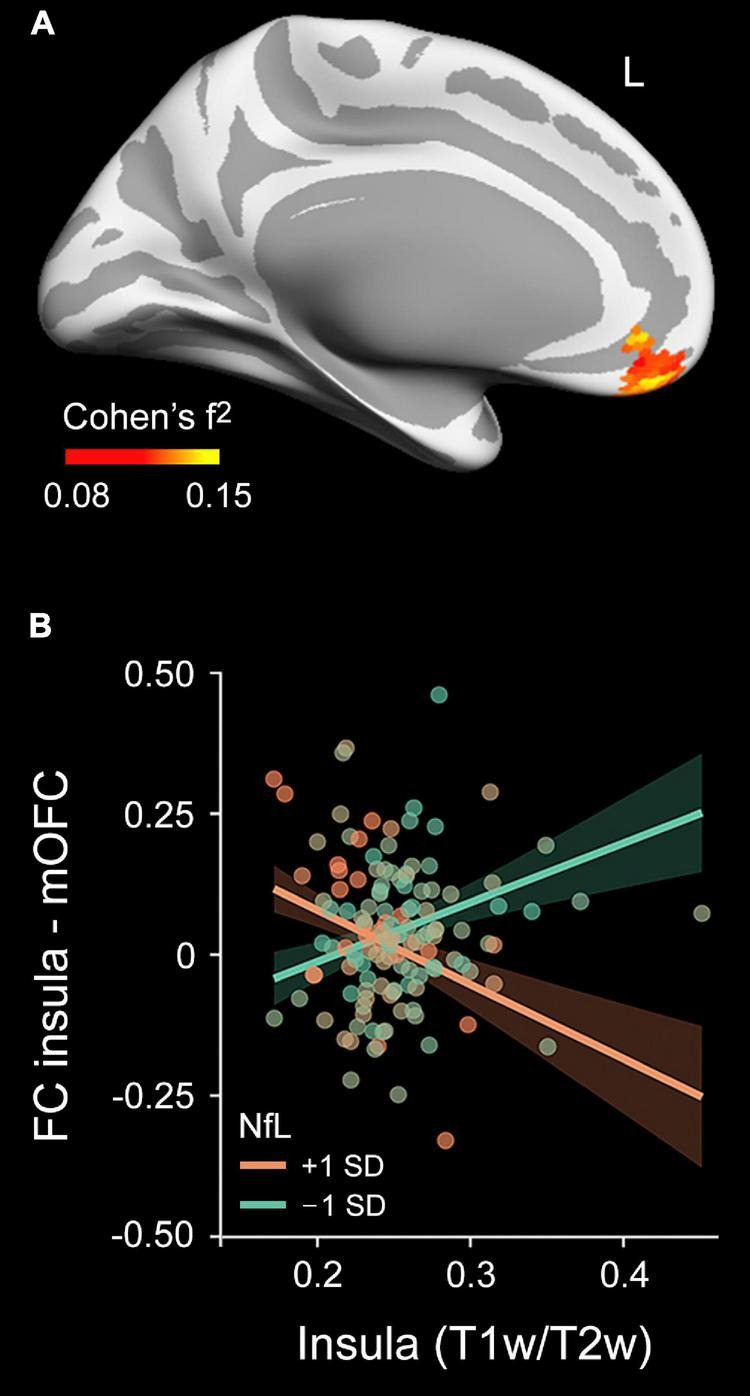
Moderating effect of plasma NfL levels on the relationship between the mean myelin content and the rs-FC pattern of the right insula. **(A)** Projected size effects on the inflated cortical surface show the moderating role of plasma NfL levels in the association between the myelin content and rs-FC pattern of the right insula. The color bar indicates the overall range of size effects (Cohen’s f^2^). Left (L). **(B)** Scatter plot for the association of the mean myelin content of the right insula with rs-FC between the right insula and left medial orbitofrontal cortex (mOFC) at 1 SD below and above the mean of plasma NfL levels, adjusted by age and sex.

We next performed rs-FC analysis using as seeds those regions derived from significant associations between plasma measurements (Aβ_1–42_ and NfL) and myelin content (regions displayed in Figures 3, 5). The interaction between plasma Aβ_1–42_ and intracortical myelin did not predict rs-FC for none of the seeds evaluated. Conversely, the multiple regression analysis performed on the rs-FC map with the right insula as seed revealed a significant two-way interaction in the left medial orbitofrontal cortex ($R_{\text{max}}^{2}$ = 0.13, *t*_max_ = 4.36, *p*_cluster_ = 0.006, *rho* = –2.22, *CI*_95%_[–2.81 to –1.63]). Figure 6A shows the spatial location of this result. *Post hoc* analyses shown in Figure 6B revealed a negative relationship between FC and myelin in individuals with higher plasma levels of NfL (i.e., the group with + 1SD) and a positive relationship in those with lower NfL levels (i.e., the group with –1SD). The Bayesian analysis yielded a BF_10_ of 304, and therefore the evidence in favor of the alternative hypothesis is considered as extreme.

### Associations of Plasma Measurements, Intracortical Myelin and Resting State-Functional Connectivity With Cognition

The ANOVAs performed to compare the sequential models revealed no significant main or interaction effects on cognition. BF_10_ ranged between 0.33 and 1. Accordingly, no evidence in favor of the null hypothesis was observed in either case.

## Discussion

We showed that plasma levels of Aβ1-42 and NfL were associated with intracortical myelin content, suggesting that they are able to track variations in myelin content in the aging neocortex. Interestingly, associations with plasma NfL were most evident in inner cortical layers, where fibers are more densely myelinated (Nave and Werner, 2014) and oligodendrogenesis is most impaired (Orthmann-Murphy et al., 2020; Xu et al., 2021). Furthermore, we found that higher concentrations of plasma NfL were associated with aberrant FC patterns of the insular cortex, a central brain hub highly vulnerable to neurodegeneration involved in affective and cognitive processes (Cauda et al., 2011). Considering that myelin undergoes significant alterations during aging and that aging is the major risk factor for AD, these results may be helpful in detecting myelin dysfunctions in aging and hypothetically serve to identify vulnerability to develop AD.

### Plasma Aβ_1–42_ Levels Are Positively Associated With Intracortical Myelin Content

Previous studies have shown that low plasma Aβ_1–42_ concentrations appear in MCI/AD patients carrying the Apolipoprotein E ε4 allele (APOE4) (Kleinschmidt et al., 2016) and in cognitively normal individuals showing abnormal CSF-amyloid status and positive amyloid PET scans (Verberk et al., 2018). Moreover, plasma Aβ_1–42_ levels have been negatively associated with WM hyperintensities and positively with global cognition, memory performance, hippocampal volume and cortical thickness of the temporal lobe (Llado-Saz et al., 2015; Poljak et al., 2016; Hilal et al., 2018), as well as with a steeper rate of subsequent cognitive decline (Rembach et al., 2014; Poljak et al., 2016; Verberk et al., 2020) and increased risk of AD (Chouraki et al., 2015; Hilal et al., 2018; de Wolf et al., 2020).

Our results revealed a positive relationship between plasma Aβ_1–42_ levels and intracortical myelin in cognitively normal older adults, linking plasma amyloid markers to cortical myelin integrity in normal aging. The association between cerebral amyloid aggregation and myelin deficits has been mainly supported by research in mouse models of AD (Desai et al., 2010; Mitew et al., 2010; Schmued et al., 2013; Chen et al., 2021). These studies showed that myelin pathology extends across regions with Aβ deposits (Mitew et al., 2010; Schmued et al., 2013), suggesting that the absence of oligodendrocytes in the plaque core may contribute to the destabilization of neuronal networks surrounding Aβ deposits (Palop and Mucke, 2010). This interpretation has been reinforced by research showing *in vivo* associations between myelin alterations and amyloid pathology in preclinical AD (Dean et al., 2017; Luo et al., 2019). In particular, lower CSF Aβ_1–42_ levels were found to be associated with lower myelin water fraction (MWF) in the WM of late myelinating brain regions (Dean et al., 2017), which are affected by AD lesions from the earliest stage (Braak and Del Tredici, 2015). Further evidence indicates that preclinical AD patients exhibit the lowest T1w/T2w ratio in the inferior parietal lobe, which continues to decrease with disease progression (Luo et al., 2019). Our results complement these findings, providing evidence that positive associations of intracortical myelin with plasmatic Aβ_1–42_ are restricted to posterior cortical regions in normal aging. Together, these results suggest that myelin breakdown in aging and preclinical AD occurs in the reverse direction in which myelination develops, from cortical association regions to subcortical WM projection tracts, and from posterior to anterior brain regions (Bartzokis, 2004).

An alternative interpretation is that the load of WM lesions, instead of Aβ deposition in plaques, could drive the relationship between plasma Aβ_1–42_ and intracortical myelin content. In line with this hypothesis, it has been reported that associations between CSF Aβ_1–42_ levels and cortical atrophy are noticeable in dementia patients but absent in cognitively normal older adults, whereas correlations between CSF Aβ_1–42_ and WM lesions are present in normal elderly individuals but lacked in dementia patients (Skoog et al., 2018). Further, if associations between plasma Aβ_1–42_ and intracortical myelin content were a reflection of the cerebral amyloid burden, this relationship should be stronger in deeper cortical layers, where the myelin content is higher (Hughes et al., 2018). This prediction is supported by evidence indicating that Aβ staining in 5×FAD transgenic mice is more intense in deeper than in superficial cortical layers. As a result, T1 and T2 values were more drastically reduced in deeper cortical regions in transgenic mice compared to wild-type mice (Spencer et al., 2013). Moreover, analysis of the laminar distribution of transmitter receptors in the human neocortex has shown that deeper layers of temporo-occipital regions concentrate a high density of kainate receptors (Palomero-Gallagher and Zilles, 2019). Given that oligodendrocytes contribute to the control of extracellular glutamate levels (Matute et al., 2001) and oligodendrogenesis is mostly impaired in the deeper cortical layers (Orthmann-Murphy et al., 2020; Xu et al., 2021), alterations in glutamate homeostasis may result in overactivation of kainate receptors and subsequent excitotoxic oligodendroglial death, especially in the innermost layers. However, our results showed that associations between plasma Aβ_1–42_ and intracortical were stronger in the outer cortical layer (at least in the right hemisphere), suggesting that plasma Aβ_1–42_-myelin associations are not likely driven by cerebral amyloid burden.

It is also worth noting that T1 relaxation rate correlates with iron concentration in the brain, showing much higher iron relaxivity values in the cortex than in other brain structures (Ogg and Steen, 1998). Accordingly, high-field MRI data from postmortem brain samples has revealed a layered organization of iron content in the cortical mantle suggestive of each region’s myeloarchitecture (Fukunaga et al., 2010). Therefore, we cannot rule out that associations between plasma measurements and intracortical myelin content are partially due to iron accumulation. Future experiments should use more sophisticated *in vivo* approaches to separate iron and myelin content in the human cortex (Shin et al., 2021) in order to reliably determine their specific effects on aging and aging-related neurodegenerative conditions.

### Plasma NfL Levels Are Negatively Associated With Intracortical Myelin Content and Abnormal Resting State-Functional Connectivity

Neurofilament light chain is one of the scaffolding proteins of the axonal cytoskeleton with important roles in axonal and dendritic branching (Yuan et al., 2017). NfL is particularly abundant in myelinated axons and is released to the periphery upon damage of the central nervous system (Gafson et al., 2020). Although plasma NfL is not a specific AD biomarker, it has been positively associated with higher risk of cognitive decline in cognitively normal older adults, MCI and AD patients (Mattsson et al., 2017; Aschenbrenner et al., 2020; Lee et al., 2022) and higher CSF NfL concentration in the early presymptomatic stages of familial AD (Preische et al., 2019) and in all stages of sporadic AD (Mattsson et al., 2017). At baseline, it has been positively related to neocortical Aβ and tau in cognitively unimpaired and impaired older adults, respectively (Chatterjee et al., 2018; Benedet et al., 2020) and negatively to hippocampal atrophy in all stages of AD (Mattsson et al., 2017; Hu et al., 2019) and to the mean cortical thickness of the whole-brain and specific brain regions in MCI and cognitively normal older adults (Lee et al., 2022). Over time, decreasing plasma NfL levels have also been related to progressive enlargement of the lateral ventricles and decreasing brain metabolism, hippocampal volume and cortical thickness especially in MCI and AD patients (Mattsson et al., 2017; Benedet et al., 2020). Consequently, plasma NfL is considered a promising and cost-effective biomarker of axonal injury in AD and a variety of neurological conditions (Khalil et al., 2018).

High blood NfL levels may further reflect myelin breakdown, presumably due to dysfunctional oligodendrocytes, which provide trophic support to axons (Mot et al., 2018). Multiple lines of evidence indirectly support this hypothesis in humans. For instance, recent experiments have revealed associations between high serum NfL levels and MRI markers of myelin damage in multiple sclerosis (Yik et al., 2022), pediatric acquired demyelinating syndrome (Simone et al., 2021), and X-linked adrenoleukodystrophy (Weinhofer et al., 2021). Our results add further support to this hypothesis, showing a negative relationship between plasma NfL levels and intracortical myelin content of the right insular cortex, a lightly myelinated cortical region that has shown increased vulnerability in aging (Hu et al., 2014), preclinical (Cantero et al., 2020) and prodromal AD (Cantero et al., 2017).

We also showed that plasma NfL levels modulated patterns of FC between the insula and medial orbitofrontal cortex, two lightly myelinated cortical areas that appeared to be vulnerable to detrimental effects of aging (Bartzokis, 2011; Vidal-Piñeiro et al., 2016). Recent evidence has linked rs-FC patterns of insular cortex and orbitofrontal cortex to apathy (Jang et al., 2021), a multi-domain syndrome associated with poor outcomes and incident dementia in normal older adults (van Dalen et al., 2018), suggesting that impaired FC of these two regions may have unfavorable implications for aging. Tract tracing studies in primates have revealed that the anterior insula has prominent connections to the orbitofrontal cortex (Flynn et al., 1999), forming a functional unit that serves in the integration of complex autonomic, cognitive and emotional processes (Morel et al., 2013), all of them manifestly affected by aging (Salthouse, 2012). We speculate that as long as plasma NfL levels are elevated, reduced myelin content of the insular cortex may affect synchronized timing of neuronal impulses leading to abnormal functional connections with the medial orbitofrontal cortex.

### Study Limitations

Results of the present study were cross-sectionally obtained, which has strengths and weaknesses that should be acknowledged. While cross-sectional studies are valuable for establishing preliminary evidence in planning advanced studies, they are not helpful for drawing predictive conclusions. As long as the study sample is representative of the overall population, cross-sectional studies are adequate for measuring the prevalence of health outcomes, understand determinants of health, and describe features of a population (Wang and Cheng, 2020). Therefore, our results cannot establish a true cause and effect relationship between T1w/T2w ratio values and plasma levels of Aβ_1–42_ and NfL, nor can they determine a temporal relationship between outcomes and risk factors. However, they could be used to generate predictive hypothesis for longitudinal studies in which T1w/T2w ratio values are used as a predictive or dependent variable.

Linking our results to AD requires caution. Brain-specific proteins are present in much lower concentrations in blood than in CSF because the blood-brain barrier prevents free passage of molecules between the CNS and blood compartments (Zetterberg and Burnham, 2019). More importantly, Aβ species are expressed in non-brain tissues and bind to a variety of blood proteins (Marcello et al., 2009), which may not reflect brain Aβ turnover/metabolism and consequently reduce the potential for monitoring Aβ pathology in the blood. Moreover, the plasma Aβ_1–42_/Aβ_1–40_ ratio has shown better concordance with cerebral amyloid load than plasma Aβ_1–42_ alone (Janelidze et al., 2016; Schindler et al., 2019), suggesting that the Aβ_1–42_/Aβ_1–40_ ratio has an added value as a pre-screening biomarker in AD.

## Conclusion

The present study showed that lower plasma Aβ_1–42_ and higher plasma NfL levels were associated with lower intracortical myelin content in temporo-parietal-occipital regions and the insula, respectively. Notably, the latter association was most evident in inner cortical layers, where axons are more heavily myelinated (Nave and Werner, 2014) and oligodendrogenesis is most impaired (Orthmann-Murphy et al., 2020; Xu et al., 2021). Plasma NfL levels further moderated the relationship between intracortical myelin content and rs-FC, likely revealing a complex inter-relationship between axonal damage, myelin breakdown and FC in aging. As myelin undergoes significant alterations in aging, the most important risk factor for AD, these results may be helpful in detecting aging-related myelin dysfunctions and potentially serve to identify vulnerability to develop AD.

## Data Availability Statement

The raw data supporting the conclusions of this article will be made available by the authors on reasonable request.

## Ethics Statement

The studies involving human participants were reviewed and approved by Ethical Committee for Clinical Research of the Junta de Andalucía. The patients/participants provided their written informed consent to participate in this study.

## Author Contributions

JLC conceived the study and wrote the manuscript. MF-A, MA, and JLC contributed to data acquisition, data analysis, and preparation of figures and tables. FZ, CM, and EC-Z performed the plasma Aβ_1–42_ and NfL determinations. All authors read and approved the final version of the manuscript.

## Conflict of Interest

The authors declare that the research was conducted in the absence of any commercial or financial relationships that could be construed as a potential conflict of interest.

## Publisher’s Note

All claims expressed in this article are solely those of the authors and do not necessarily represent those of their affiliated organizations, or those of the publisher, the editors and the reviewers. Any product that may be evaluated in this article, or claim that may be made by its manufacturer, is not guaranteed or endorsed by the publisher.

## Footnotes

1
https://surfer.nmr.mgh.harvard.edu/

2
https://afni.nimh.nih.gov/afni

3
https://www.psychologie.hhu.de/arbeitsgruppen/allgemeine-psychologie-und-arbeitspsychologie/gpower.html

4
https://www.math.mcgill.ca/keith/surfstat/

5
https://jasp-stats.org/
