# Supplementary material for: Linking Plasma Amyloid Beta and Neurofilament Light Chain to Intracortical Myelin Content in Cognitively Normal Older Adults
Source: Front Aging Neurosci. 2022 Jun 17;14:896848. doi: 10.3389/fnagi.2022.896848 (PMC9247578; doi:10.3389/fnagi.2022.896848)
Supplement: Supplementary file 1 [file Data_Sheet_1.PDF]

Supplementary Material

Linking plasma amyloid beta and neurofilament light chain to intracortical myelin content in cognitively normal older adults

Marina Fernandez-Alvarez[1,2], Mercedes Atienza[1,2], Fatima Zallo[3], Carlos Matute[2,3], Estibaliz Capetillo-Zarate[2,3,4], Jose L. Cantero[1,2*]

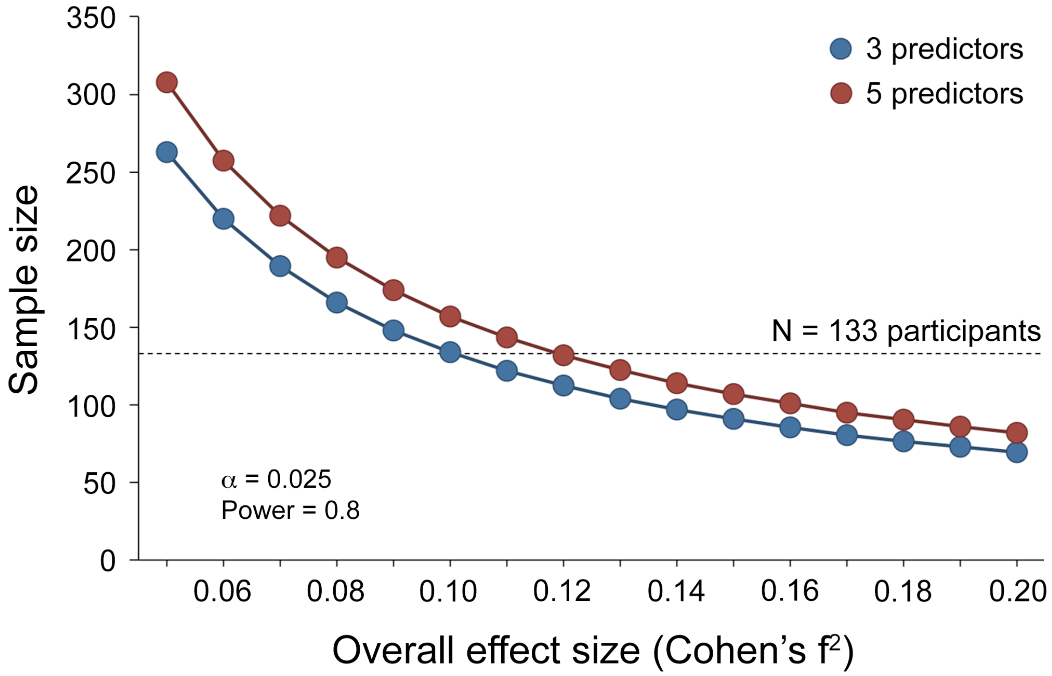

Figure SM1. Power analysis to estimate the sample size when the model included 3 (blue circles) or 5 predictors (red circles). The plot shows the estimated sample size to achieve statistical power of 80% given a 0.025 two-sided significance level (α) and an overall Cohen's effect size (f^2) ranging from 0.05 to 0.2.

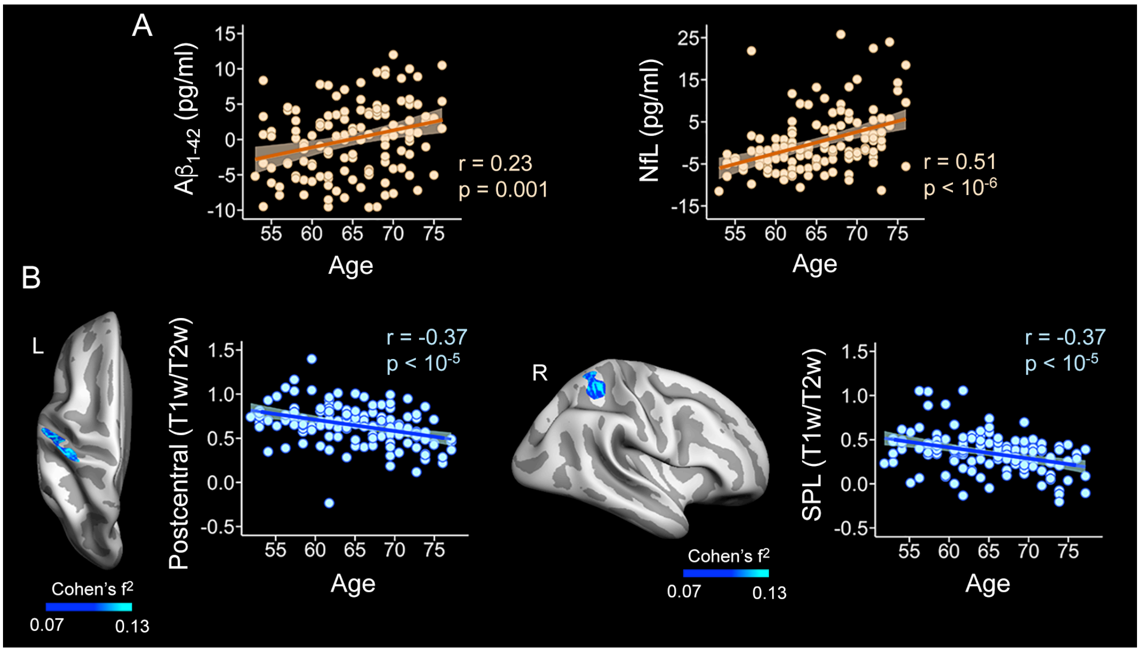

Figure SM2. (A) Correlations between plasma measurements ($A\beta_{1\text{-}42}$ and NfL levels) and age, adjusted by sex. **(B)**. Surface-based regression analyses correlating intracortical myelin content (i.e., T1w/T2w values) with age, adjusted by sex. Scatter plots show correlations between age and the mean myelin content of the significant cluster, adjusted by sex. The color bar indicates the range of size effects (Cohen's f^2). Left (L) and right (R). SPL: superior parietal lobe.
